# Epidemiological Trends, Public Health Challenges, and Strategic Control Priorities of Dengue in Bangladesh (2000—2024): A Narrative Review

**DOI:** 10.1002/hsr2.72747

**Published:** 2026-07-04

**Authors:** Md. Jubayer Hossain, Nishat Afrin Mim, Nargees Akter, Soniya Akter Sony, Ekramul Haque Saikat, Md. Al‐Amin Hossain, Nusrat Parvin, M. R. Evan, Jayma Jannat Juma, Progga Parmita Roy, Md. Fakhrul Islam Maruf, Rayhan Islam, Abrar Labib, Zayeda Akter Shatabde, Manisha Das, Md Amanulla

**Affiliations:** ^1^ Center for Health Innovation, Research Action and Learning—Bangladesh (CHIRAL Bangladesh Dhaka Bangladesh; ^2^ Department of Public Health Daffodil International University Dhaka Bangladesh; ^3^ Geospatial Health Research Group Center for Health Innovation, Research Action, and Learning—Bangladesh (CHIRAL Bangladesh) Dhaka Bangladesh; ^4^ Department of Public Health and Informatics Jahangirnagar University, Savar Dhaka Bangladesh; ^5^ Department of Geography and Environmental Studies University of Chittagong Chittagong Bangladesh; ^6^ Population Health Studies Division Center for Health Innovation, Research Action, and Learning—Bangladesh (CHIRAL Bangladesh) Dhaka Bangladesh; ^7^ aiHealth Lab, Center for Health Innovation, Research Action, and Learning—Bangladesh (CHIRAL Bangladesh) Dhaka Bangladesh; ^8^ Department of Applied Mathematics University of Rajshahi Rajshahi Bangladesh; ^9^ Department of Biomedical Engineering and Public Health World University of Bangladesh Dhaka Bangladesh; ^10^ Department of Occupational and Environmental Health National Institute of Preventive and Social Medicine Dhaka Bangladesh; ^11^ Dhaka Medical College Dhaka Bangladesh; ^12^ Department of Physiotherapy and Rehabilitation Jashore University of Science and Technology, Bangladesh Jashore; ^13^ Department of Civil and Environmental Engineering Islamic University of Technology Gazipur Bangladesh; ^14^ Big Bioinformatics Lab, Center for Health Innovation, Research Action, and Learning—Bangladesh (CHIRAL Bangladesh) Dhaka Bangladesh; ^15^ Department of Microbiology Noakhali Science and Technology University Noakhali Bangladesh; ^16^ Disaster Management & Resilience Bangladesh University of Professionals Dhaka Bangladesh

**Keywords:** Bangladesh, dengue, epidemiology, insecticide resistance, one health, outbreaks, public health response, vector control

## Abstract

**Background and Aims:**

Dengue has evolved into a major public health crisis in Bangladesh, transitioning from sporadic outbreaks to endemic transmission with increasing frequency and severity. The unprecedented 2023 epidemic recorded more deaths than the cumulative total of the previous two decades, highlighting the urgent need for a comprehensive synthesis of epidemiological trends and control challenges.

**Methods:**

This narrative review synthesizes published literature, surveillance reports, and national health data from 2000 to 2024 to examine the evolving epidemiology, clinical characteristics, transmission dynamics, and public health responses related to dengue in Bangladesh.

**Results:**

Epidemiological analysis reveals a dramatic rise in incidence and mortality, with widespread geographic expansion beyond Dhaka into southern and rural districts and a shift toward earlier seasonal peaks. Serotype transitions, particularly the dominance of DENV‐3 followed by DENV‐2—likely intensified disease severity through secondary infections. High case fatality rates were observed among females and older adults, with a substantial proportion of deaths occurring within 24 h of hospitalization, suggesting critical gaps in care‐seeking and clinical management. Transmission dynamics are shaped by interactions between Aedes vector ecology, climate change, rapid urbanization, human mobility, and extensive insecticide resistance to pyrethroids. Public health responses remain constrained by passive surveillance, limited vector control efficacy, healthcare system strain, and insufficient community engagement.

**Conclusions:**

The dengue burden of Bangladesh underscores the need for integrated and adaptive control strategies. Strengthening multi‐domain surveillance (epidemiological, entomological, genomic), implementing resistance‐aware Integrated Vector Management incorporating novel approaches such as Wolbachia, enhancing healthcare readiness, promoting community‐driven behavioral interventions, integrating climate adaptation measures, and advancing vaccine and therapeutic research within a One Health framework are critical for sustainable dengue prevention and control.

## 1 Introduction

Dengue virus (DENV) infection represents one of the most rapidly spreading mosquito‐borne viral diseases globally, posing a substantial and growing threat to public health, particularly in tropical and subtropical regions [1, 2, 3]. The World Health Organization (WHO) estimates that more than 4 billion people in over 100 countries are now at risk, with an estimated 100 to 400 million infections occurring annually, concentrated heavily in South and Southeast Asia, and the Western Pacific region accounts for 75% of the world's dengue burden [3, 4, 5]. Global confirmed cases reported to WHO surged dramatically from just over half a million in 2000 to 2.4 million in 2010 and over 5.2 million in 2019, indicating a significant intensification and geographic expansion of transmission [4, 6, 7]. The world confronted its largest recorded burden in 2024, with over 14.1 million cases reported globally [8].

Bangladesh, a densely populated South Asian nation, has a history with dengue dating back to the 1960s when it was known locally as “Dacca fever” [9, 10]. However, the contemporary dengue problem began with its re‐emergence and the first officially documented outbreak in 2000, which resulted in 5551 reported cases and 93 deaths [1, 11, 12, 13, 14]. Since then, dengue has become firmly established as an endemic disease in the country, with outbreaks occurring regularly, typically coinciding with the monsoon and post‐monsoon seasons [1].

In recent years, this situation has escalated significantly. Bangladesh has experienced successive major dengue outbreaks with increasing frequency and magnitude, particularly since 2018 [15]. The epidemics of 2019 (over 101,354 cases, 179 deaths) and 2022 (over 61,732 cases, 281 deaths) were severe, but the 2023 outbreak surpassed all previous records, constituting the largest and deadliest dengue epidemic in the nation's history [4]. Official figures reported over 321,179 hospitalizations and 1705 deaths in 2023 alone. In 2024, there was another fatal dengue outbreak in Bangladesh, with over 100,000 illnesses and 575 fatalities [16]. Alarmingly, the number of deaths in 2023 and 2024 surpassed the cumulative death toll from the preceding 22 years (2000–2022), and over 82% of total reported cases and 69% of deaths between 2000 and 2022 occurred in the 5 years from 2018 to 2022 [15]. This escalating crisis underscores the urgent need for a comprehensive understanding of the factors driving transmission and the effectiveness often current control measures.

This narrative review aimed to synthesize the available evidence on the evolving epidemiology, clinical challenges, complex transmission dynamics, public health responses, and existing interventions related to dengue in Bangladesh, focusing on the period from 2000 to the present. By examining recent epidemiological trends, analyzing the impact of factors such as climate change and insecticide resistance, evaluating the strengths and weaknesses of surveillance and control efforts, and drawing lessons from recent devastating outbreaks, this review seeks to identify critical research gaps and propose evidence‐based future directions for dengue prevention and control. The objective was to provide an authoritative overview suitable for researchers, public health professionals, clinicians, and policymakers engaged in combating this major public health threat in Bangladesh and other endemic regions.

## 2 The Evolving Epidemiological Landscape (2000–2024)

The epidemiology of dengue in Bangladesh has undergone a dramatic transformation since its re‐emergence in 2000. Initially characterized by sporadic outbreaks primarily confined to urban centers, the disease pattern has shifted towards hyperendemicity, with larger, more frequent epidemics exhibiting significant changes in geographic distribution, seasonality, and serotype dominance, culminating in an unprecedented national crisis in 2023.

### 2.1 Temporal Trends in Incidence and Mortality

The documented burden of dengue in Bangladesh has increased exponentially over the past two decades (Table 1). From January 2000 to March 2024, official records indicate a total of 565,438 dengue cases and 2587 fatalities. However, these figures, primarily derived from hospital‐based surveillance, likely represent a significant underestimation of the true burden, as many mild, subclinical, or asymptomatic infections go unreported, and surveillance systems face limitations in coverage and reporting completeness [4]. A recent modeling study estimated as many as 2.4 million new infections occur annually [11, 18].

**TABLE 1:** Dengue burden and serotype dynamics in Bangladesh (selected years 2000–2024).

Year	Reported cases	Reported deaths	Case fatality rate (CFR %)	Dominant serotype(s) (if available)	Source snippets
2000	5551	93	1.67	DENV‐3	[1]
2002	6232	58	0.93	DENV‐3	[1]
2019	101,354	179	0.17	DENV‐3	[1]
2020	1193	3	0.25	DENV‐3	[1]
2021	28,429	105	0.37	DENV‐3	[1]
2022	61,732	281	0.45	DENV‐2 (emerging), DENV‐4 (detected)	[1]
2023	321,179	1705	0.53	DENV‐2 (70.2%), DENV‐3 (23.9%)	[4]
2024	101,214	575	0.57	DENV‐2 (70.2%), DENV‐3 (20.2%), DENV‐2 + 3 (8%)	[17]
Total (Jan 2000–Mar 2024)	565,438	2587	~0.46	DENV‐3 (57%), DENV‐2 (30%) overall	[4]

*Note:* Case and death counts, CFR, and dominant serotypes can vary slightly between sources/reports depending on reporting period and methodology. This table synthesizes representative data from the provided snippets.

The major outbreak years punctuate this period, demonstrating a clear trend of escalating intensity. Following the initial 2000 outbreak (5551 cases, 93 deaths), significant epidemics occurred in 2002 (6232 cases, 58 deaths) and periodically thereafter. The scale shifted dramatically in recent years: 2019 witnessed 101,354 hospitalized cases and 179 deaths; 2022 saw over 61,732 cases and a then‐record 281 deaths [1]; and 2023 shattered all previous records with over 321,179 cases and 1705 deaths reported. The sheer scale of the 2023 epidemic is underscored by the fact that the number of cases reported was 1.3 times the total recorded in the previous 23 years combined, and the death toll was twice the cumulative fatalities over the same period [15].

**FIGURE 1:**
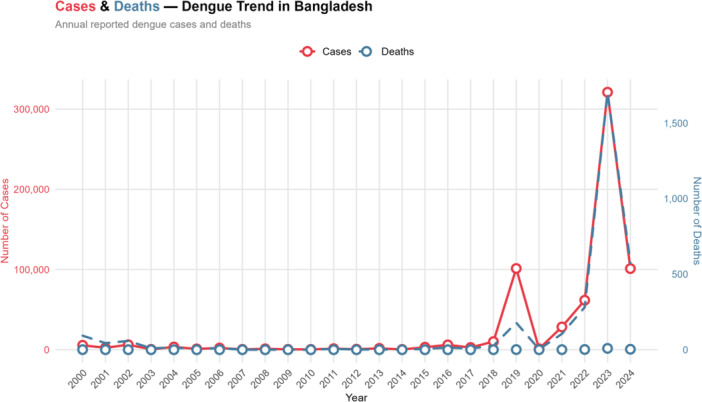
Annual Trends of Reported Dengue Cases and Deaths in Bangladesh (2000–2024). This dual‐axis line graph illustrates the long‐term epidemiological trajectory of dengue in Bangladesh. The red solid line (left *Y*‐axis) represents the number of annual hospitalizations, while the blue dashed line (right *Y*‐axis) tracks annual fatalities. The visualization highlights the dramatic escalation in 2023, where both cases (321,179) and deaths (1705) reached unprecedented historical peaks. Data sourced from the Directorate General of Health Services (DGHS) and official IEDCR reports. IEDCR, Institute of Epidemiology, Disease Control and Research.

**FIGURE 2:**
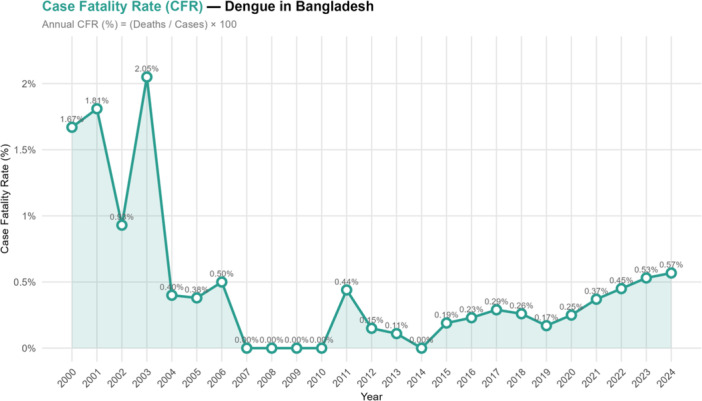
Annual Case Fatality Rate (CFR) Trends for Dengue in Bangladesh (2000–2024). This trend line chart displays the annual CFR (deaths/total cases × 100). While early outbreaks (2000–2003) showed high CFR volatility due to low case numbers, a sustained and concerning upward trend in mortality severity is observed from 2019 onwards, culminating in a record high of 0.57% in 2024, suggesting increasing disease severity linked to serotype shifts (DENV‐2 dominance) and healthcare system strain. Data sourced from DGHS. DGHS, Directorate General of Health Services.

The Case Fatality Rate (CFR) also showed trends. While fluctuating over the years (ranging from 0% in some years with low case numbers to over 2% in 2003), the CFR has notably increased during recent large outbreaks [1]. The mean CFR over the 23 years prior to 2023 was approximately 0.35% [15]. In contrast, the CFR during the massive 2023 outbreak was significantly higher, reported between 0.47% (as of Aug 2023) [9] and 0.53% (for the full year) [15]. This rate placed Bangladesh among the countries with the highest dengue CFR globally in 2023, surpassing nations with even larger case numbers like Brazil [19]. The combination of exponentially increasing case numbers and increasing CFR points towards a deepening crisis. This suggests that factors beyond just increased transmission efficiency are at play, potentially including shifts towards more virulent viral strains, increased population susceptibility to severe disease due to previous exposures, or a healthcare system increasingly strained beyond its capacity to effectively manage the surge of critically ill patients [15]. The elevated CFR, particularly during the largest recorded outbreak, signals potential systemic failures in either preventing severe disease progression or providing timely and adequate critical care (Figures 1 and 2).

### 2.2 Shifting Geographical Distribution

A defining feature of the evolution of dengue in Bangladesh is its dramatic geographical expansion. Historically, dengue transmission was highly concentrated in the capital city, Dhaka, and to some extent, other major urban centers like Chittagong and Khulna [15]. Seroprevalence studies conducted between 2014 and 2016 confirmed this pattern, finding 36% to 85% seropositivity in Dhaka and 84% to 88% in Chittagong compared to only 3% in some northern rural areas [15, 18]. Dhaka consistently accounted for the majority of reported cases in the early years and even into the late 2010s [15, 20].

**FIGURE 3:**
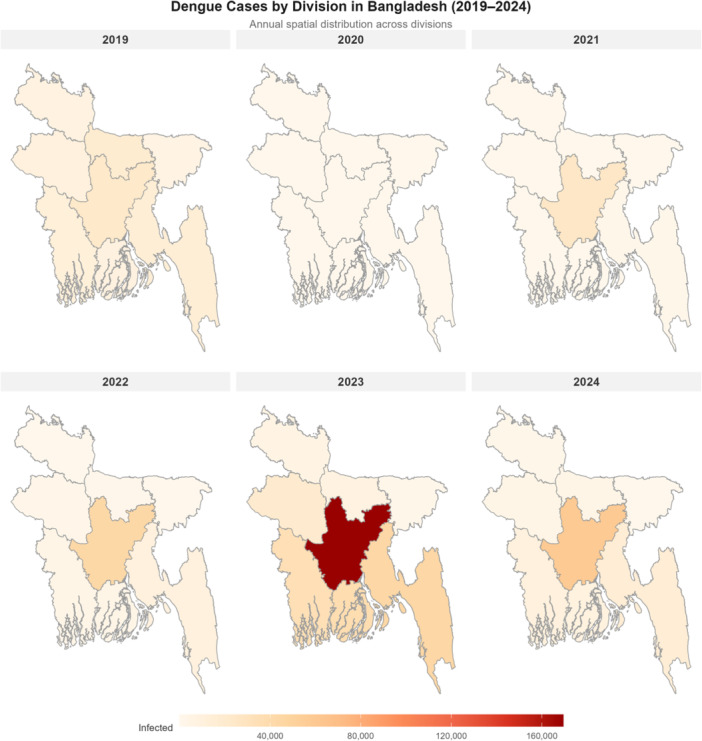
Annual spatial distribution of dengue cases by division in Bangladesh (2019–2024). These annual choropleth heat maps depict the geographic expansion of dengue infections across the eight administrative divisions of Bangladesh. Color intensity (light to dark) represents case burden from low to high. The progression from 2019 to 2023 demonstrates clear diffusion of dengue transmission from the primary urban epicenter of Dhaka Division to a nationwide hyperendemic state, with substantial case burdens emerging in the southern divisions of Khulna and Barishal and the southeastern division of Chattogram by 2023. Data sourced from DGHS/IEDCR. DGHS, Directorate General of Health Services; IEDCR, Institute of Epidemiology, Disease Control and Research.

**FIGURE 4:**
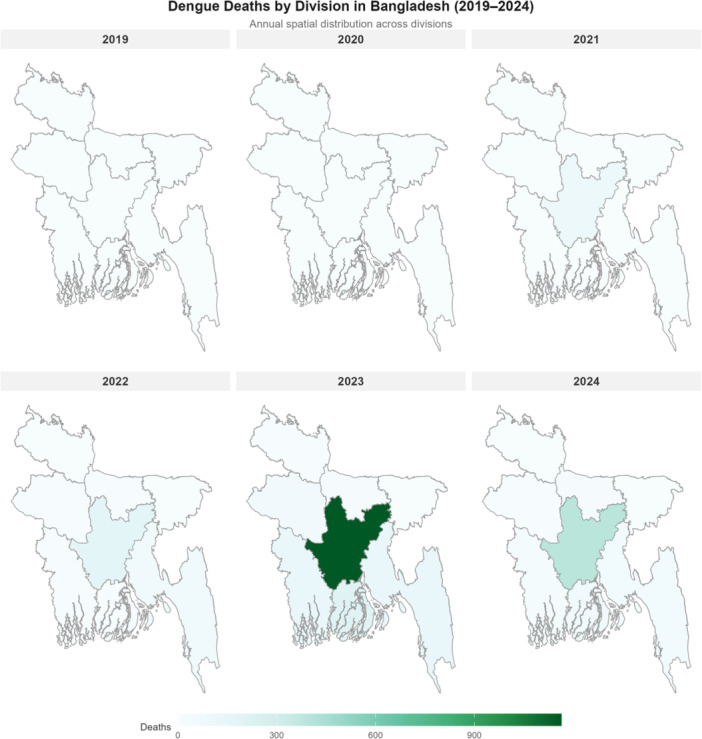
Annual spatial distribution of dengue deaths by division in Bangladesh (2019–2024). These choropleth maps depict the spatial distribution of dengue‐related mortality across Bangladesh's administrative divisions. Color intensity represents mortality burden from low (light) to high (dark). Mortality intensity shows a significant increase in geographic scope over the study period, with the 2023 map highlighting a critical concentration of fatalities in Dhaka Division while simultaneously reflecting the emergence of mortality clusters in previously low‐risk southern and southeastern divisions. Data sourced from DGHS/IEDCR. DGHS, Directorate General of Health Services; IEDCR, Institute of Epidemiology, Disease Control and Research.

However, the landscape changed rapidly. The major outbreaks of 2019 and particularly 2023 witnessed a significant diffusion of dengue transmission across the country [4]. By 2023, cases were reported from all 64 districts of Bangladesh [21]. Notably, during the 2019 and 2023 epidemics, approximately 60% of cases occurred in regions previously considered non‐endemic or low‐risk. New hotspots emerged, particularly in the southern divisions of Khulna and Barishal, and the southeastern division of Chattogram, which reported substantial proportions of the national case burden in 2023 [4]. Analysis of the 2023 outbreak revealed a significantly higher incidence per 1000 population in the divisions south of Dhaka compared to the northern divisions (2.30 vs. 0.50) [15]. While Dhaka city remained a major focus, its relative contribution to the national total decreased, accounting for roughly 53% of cases by August 2023 but only 35% for the entire year, indicating widespread transmission elsewhere [9]. Persistent hotspots were identified in districts like Bagerhat, Barisal, and Faridpur [22]. The spatial distribution of dengue cases and deaths across Bangladesh's eight divisions from 2019 to 2024 is illustrated in Figures 3 and 4. The geographic expansion pattern and contributing factors have been further characterized in a recent evidence synthesis.

Furthermore, the traditional view of dengue as an exclusively urban disease is being challenged, with increasing evidence of significant transmission occurring in rural communities. This expansion into rural areas may be facilitated by the secondary vector, Aedes albopictus, which is more adapted to non‐urban environments compared to the primarily urban Ae. aegypti [15]. The observed rapid geographic spread, especially the seeding of outbreaks in previously unaffected rural areas and the emergence of new regional hotspots, cannot be explained by localized factors alone. It strongly suggests that increased human mobility and connectivity across the country, potentially amplified during major festivals where large numbers travel from urban centers to rural homes, are playing a crucial role in disseminating the virus [4, 15]. Concurrently, climate change and environmental modifications may be creating more suitable conditions for vector survival and proliferation in these new areas, overwhelming control efforts that were historically focused on Dhaka [23].

### 2.3 Changing Seasonality and Temporal Patterns

Dengue transmission in Bangladesh has traditionally followed a distinct seasonal pattern that is closely linked to the monsoon climate. Cases typically begin to rise with the onset of the rainy season around May or June, peak during the late monsoon and early post‐monsoon period (historically September to November), and decline during the drier winter months [24]. However, recent years, particularly in 2023, have shown deviations from this established pattern. The 2023 outbreak was characterized by an unusually early and sharp surge. Incidence began increasing significantly as early as May, surged rapidly from late June, and appeared to peak earlier than historically observed, around August and September, rather than later in the post‐monsoon period [9]. This contrasts with 2022, where the peak occurred later, in October [25]. Some analyses suggest a general shift in the peak month from August towards September in recent years [1, 26]. Furthermore, the 2023 season started with an unusually high number of cases in January, potentially indicating sustained transmission from the previous year's late surge [27].

Several factors are proposed to explain these temporal shifts. Unusual rainfall patterns, such as heavy pre‐monsoon rains or extended monsoon periods, can create abundant mosquito breeding sites earlier or later in the year [9]. Higher ambient temperatures, potentially linked to climate change, can accelerate mosquito development and viral replication, shortening the transmission cycle [23]. The timing of major holidays involving mass travel (e.g., Eid festivals) might also influence the timing and spread of outbreaks [15, 28, 29]. The observed alterations in seasonality imply that relying solely on historical monsoon timing to guide surveillance and control activities is becoming increasingly inadequate. Climate variability and potentially other ecological or behavioral factors appear to be disrupting the established transmission cycles, demanding more dynamic, data‐driven approaches to predict and respond to outbreaks throughout the year [4].

### 2.4 Serotype Dynamics and Implications

Bangladesh is known to have co‐circulation of all four dengue virus serotypes (DENV‐1, DENV‐2, DENV‐3, DENV‐4), although their prevalence has varied significantly over time [4] (Table 1). Understanding these dynamics is crucial, as infection with one serotype provides long‐lasting immunity only to that specific type, while subsequent infection with a different (heterologous) serotype significantly increases the risk of developing severe dengue (DHF/DSS) through mechanisms like antibody‐dependent enhancement (ADE) [1]. The history of dengue in Bangladesh since 2000 has been marked by shifts in dominant serotypes. Early outbreaks involved DENV‐3 [1, 30, 31, 32]. A significant shift occurred with the emergence and subsequent dominance of DENV‐3 starting in 2017 [33], which became the predominant serotype during the large 2019 outbreak and continued through 2021 [1, 34]. This was followed by another major shift, with DENV‐2 re‐emerging strongly and becoming the primary circulating serotype identified in the 2022 and the unprecedented 2023 outbreaks. DENV‐3 remained a significant co‐circulating serotype in 2023 [9]. Additionally, DENV‐4, previously rare, was detected or reintroduced in 2022–2023 [35, 36]. In 2023, serotyping data indicated DENV‐2 was responsible for 70.2% of cases, DENV‐3 for 23.9%, DENV‐1 for 3.3%, DENV‐4 for 0.4%, and 2.2% DENV‐2/3 co‐infections [26]. A systematic review covering 2000–2024 found DENV‐3 to be the most prevalent serotype overall (57% of isolates), followed by DENV‐2 (30%), DENV‐1 (11%), and DENV‐4 (< 1%), with genotype DENV3‐I being the most commonly identified [4]. This sequential pattern of different serotypes dominating successive outbreaks has profound public health implications. The period of DENV‐3 dominance (2017–2021) likely left a large proportion of the population immune to DENV‐3 but susceptible to other serotypes. The subsequent surge of DENV‐2 in 2022‐2023 therefore encountered a population highly vulnerable to secondary heterologous infections. This epidemiological “priming” effect is strongly suspected to be a major contributing factor to the unusually high severity, hospitalizations, and mortality observed during the 2023 outbreak [9]. It underscores the critical importance of robust, real‐time serotype surveillance to understand population immunity profiles and anticipate the potential severity of impending outbreaks.

### 2.5 Demographic Variations

Analysis of demographic data reveals that specific groups are disproportionately affected by dengue infection and mortality. While dengue historically affected adults more frequently in early outbreaks [1, 37], recent data indicate a broad impact across age groups. In the 2023 outbreak, the highest frequency of cases was reported among young adults aged 19–29 years (28.7%) [38], with the 16–35 year age group accounting for half of all cases [26]. However, mortality risk appears concentrated at the extremes of age. The highest CFR in 2023 was reported among older adults (> 60 years), with a CFR of 1.87% [9]. In one study, it also indicates a very high CFR (potentially up to 12%) among young children (0–10 years), although this specific figure warrants cautious interpretation and further verification [4].

Gender disparities are also evident and present a paradoxical pattern. Consistently, more dengue cases are reported among males [1, 30]. This may reflect differences in exposure patterns related to work or outdoor activities. However, a starkly contrasting trend is observed for mortality. Fatalities are disproportionately higher among females, who accounted for 70% of dengue deaths in recent analyses [4]. The overall CFR for females (e.g., 0.72% as of Aug 2023) was more than double that for males (0.32%), with a particularly pronounced difference in the 21–40 age group, where female CFR was four times higher than male CFR [9]. This significant discrepancy between higher male incidence and higher female mortality cannot be explained by infection rates alone. It strongly suggests the influence of other factors, potentially including gender‐related differences in healthcare‐seeking behavior (e.g., delays in seeking care due to domestic responsibilities), differential access to or quality of hospital care, underlying physiological differences in response to severe dengue, or potential biases in reporting or diagnosis [39]. This highlights a critical area requiring further investigation to understand and address potential health inequities contributing to higher female mortality from dengue.

## 3 Clinical Challenges and Disease Severity

The clinical management of dengue in Bangladesh presents significant challenges due to the broad spectrum of disease manifestations, the potential for rapid progression to severe forms, diagnostic limitations, and factors contributing to high mortality, particularly evident during large outbreaks.

### 3.1 Spectrum of Clinical Presentations

Dengue infection manifests across a wide clinical spectrum. A large proportion of infections are asymptomatic or result in a mild, undifferentiated febrile illness [4, 38]. Symptomatic cases typically present as classical Dengue Fever (DF), characterized by the abrupt onset of high fever (often 39°C–40°C or 104 °F), severe headache (often retro‐orbital), muscle pain (myalgia), joint pain (arthralgia), nausea, vomiting, and sometimes a characteristic rash [4]. Other common symptoms reported in various outbreaks in Bangladesh include fatigue, anorexia, abdominal pain, and swollen glands [4, 40, 41]. In the 2023 outbreak, fever remained nearly universal (99%), but myalgia, anorexia, and fatigue were also highly prevalent (86%), headache, malaise were (81%), hemorrhage (74%), body ache (71%), diarrhea, vomiting were (65%) while rash was reported less commonly (< 10%) [4]. Furthermore, the late‐stage atypical clinical presentations are increasingly recognized, including severe diarrhea, peripheral edema, and neurological manifestations, which can complicate diagnosis and management based solely on classical symptoms [39].

A critical concern is the progression to severe dengue, primarily Dengue Hemorrhagic Fever (DHF) and Dengue Shock Syndrome (DSS), which are life‐threatening complications characterized by plasma leakage, fluid accumulation, respiratory distress, severe bleeding, or organ impairment [42]. While most symptomatic cases are mild to moderate, a significant minority develop severe disease [4].

### 3.2 Severe Dengue and Mortality Factors

The risk of developing severe dengue is significantly elevated in individuals experiencing a secondary infection with a DENV serotype different from their primary infection [1]. This phenomenon, often attributed to ADE, where pre‐existing antibodies enhance viral uptake into immune cells, is a major driver of severity in hyperendemic regions like Bangladesh, where multiple serotypes co‐circulate and sequential infections are common. The serotype shifts observed in Bangladesh, particularly the DENV‐2 surge following DENV‐3 dominance, created conditions ripe for severe secondary infections during the 2023 outbreak [36, 43].

A particularly alarming finding from the 2023 epidemic was the extraordinarily high proportion of deaths (67.4%, *n* = 1015 out of 1705) occurring within the first 24 h of hospital admission. This statistic points towards profound issues in the continuum of care. It may reflect delays in patients seeking medical attention until they are critically ill, possibly due to a lack of awareness of warning signs, barriers to accessing care (transport, cost), or underestimation of disease severity at the community level. Alternatively, or concurrently, it could indicate that healthcare facilities, especially when overwhelmed during peak outbreak periods or in newly affected areas with limited resources, are unable to provide the rapid, intensive stabilization and management required for patients presenting with advanced DHF/DSS [15]. This rapid mortality upon reaching care signifies a critical breakdown, either in timely access or in immediate healthcare response capacity.

Other factors contributing to mortality include demographic variables, with higher CFR observed in females [4]. Underlying comorbidities likely also play a role. Additionally, some research suggests potential interactions with prior COVID‐19 infection, possibly through sustained immune dysregulation, might influence dengue severity, although this requires further investigation [19, 44, 45].

### 3.3 Diagnostic Approaches

Diagnosis of dengue in Bangladesh primarily relies on clinical presentation combined with laboratory confirmation. The most commonly used laboratory methods are rapid diagnostic tests (RDTs) detecting the DENV non‐structural protein 1 (NS1) antigen, and serological tests detecting IgM and IgG antibodies [46]. NS1 antigen is detectable early in the illness (first ~5–7 days), making it valuable for early confirmation [46, 47]. IgM antibodies typically appear around Day 4 and indicate a recent infection, while IgG antibodies appear later and can indicate past or current infection (often rising rapidly in secondary infections) [47]. Reverse transcription‐polymerase chain reaction for detecting viral RNA is the most specific method and can identify the serotype [48], but it is less frequently used, likely due to higher cost, technical requirements, and limited availability outside specialized centers [49]. The Directorate General of Health Services (DGHS) distributes NS1 RDT kits to health facilities across the country to support diagnosis [9].

While these methods are essential, they have limitations. The sensitivity of NS1 and IgM tests varies depending on the timing of sample collection relative to symptom onset. Cross‐reactivity with other flaviviruses can occur with serological tests. Crucially, the heavy reliance on NS1 RDTs for routine diagnosis [4], while facilitating early case detection, provides no information on the infecting serotype or whether the infection is primary or secondary. This lack of routine serotype data hinders real‐time surveillance of viral dynamics and population immunity, making assessing the risk of severe outbreaks driven by serotype shifts or secondary infections difficult [25, 50, 51]. Furthermore, reliance on hospital‐based testing means that many milder cases managed in the community are likely missed by the official surveillance system, contributing to the underestimation of the true disease burden [28]. The potential for misdiagnosis with other common febrile illnesses like influenza, especially when presenting with overlapping symptoms, also exists [26].

## 4 Transmission Dynamics and Eco‐Social Drivers

Dengue transmission in Bangladesh is a complex interplay of vector ecology, environmental and climatic conditions, and a range of socio‐demographic and behavioral factors, further complicated by the challenge of insecticide resistance.

### 4.1 Vector Ecology and Distribution

Two Aedes mosquito species are primarily responsible for DENV transmission in Bangladesh: Aedes aegypti and Aedes albopictus [52, 53, 54, 55]. Ae. aegypti is considered the principal vector, particularly in urban environments. It is highly anthropophilic, prefers to breed in artificial containers in and around human dwellings (indoor breeder), and feeds primarily during the daytime [56, 57]. Ae. albopictus, often referred to as the Asian tiger mosquito [58], is generally considered a secondary vector but plays an increasingly recognized role [52]. It is more adaptable, capable of utilizing both artificial and natural containers (e.g., tree holes, bamboo stumps), often breeds outdoors or in peri‐domestic settings, and may exhibit more opportunistic feeding behavior, including biting outdoors and potentially at night [26, 55, 59]. The presence and adaptability of Ae. albopictus are thought to be significant factors enabling dengue transmission in rural and semi‐urban areas where Ae. aegypti may be less prevalent [15]. Studies in Dhaka have found Ae. aegypti dominating indoor collections, while Ae. albopictus can be more common in outdoor [60].

Both species utilize a wide variety of water‐holding containers as breeding sites. Common examples identified in Bangladesh include discarded tires, plastic drums and barrels used for water storage, plastic buckets and containers, flower pots and vases, earthen jars, concrete tanks, construction site debris, and natural sites like tree holes [60, 61]. Research highlights the concept of “key containers” a limited number of container types that are responsible for producing the majority of adult mosquitoes in a given area [55, 60, 61, 62]. Identifying and targeting these key containers (e.g., water storage tanks/drums, tires, earthen jars in some studies) is crucial for efficient larval source management (LSM) [55, 60]. Vector density surveys, particularly pre‐monsoon Aedes surveys conducted by the Ministry of Health and Family Welfare of Bangladesh, often reveal high densities of mosquito larvae and potential breeding sites, especially in Dhaka, signaling high transmission risk ahead of the peak season [9]. Ae. albopictus has demonstrated mechanisms for surviving unfavorable dry seasons, such as desiccation‐resistant eggs laid in habitats like tree holes, which can contribute to the persistence of vector populations year‐round [63].

### 4.2 Environmental and Climatic Factors

Climatic conditions exert a profound influence on dengue transmission cycles by affecting mosquito biology, viral replication, and vector–human interactions [64]. Key variables include:

Temperature: Higher temperatures generally accelerate mosquito larval development, shorten the time required for adult emergence, increase biting frequency, and reduce the extrinsic incubation period (the time for the virus to replicate within the mosquito to become infectious) [64]. However, extreme heat can reduce mosquito survival [65, 66, 67]. Studies in Bangladesh consistently show a positive association between temperature (mean, max, or min) and dengue incidence [15, 23, 68].

Rainfall: Rainfall plays a dual role. Moderate rainfall creates breeding habitats by filling containers [64]. However, very heavy rainfall can wash away larvae and pupae from breeding sites [67, 69, 70]. The relationship between rainfall and dengue incidence in Bangladesh appears complex, with some studies finding positive associations [20, 71]. Others' negative [15, 23, 72], and evidence suggesting time‐dependent effects where rainfall impact varies across seasons [9, 73]. Unusual rainfall patterns (early, late, or episodic heavy rain) have been linked to outbreak timing and intensity [9, 72, 73].

Humidity: Relative humidity influences mosquito longevity, activity, and egg survival [64]. Higher humidity generally favors mosquito survival. Studies in Bangladesh have found positive associations between relative humidity and dengue cases [15, 73], although one modeling study suggested it might be redundant if other variables are included [74].

Modeling studies incorporating meteorological data in Bangladesh have confirmed these associations, often highlighting lag effects, meaning that changes in weather conditions impact dengue incidence weeks or months later [74]. For instance, one study found weather effects were strongest at a lag of 4 months [75], while another identified precipitation as most impactful at 8‐month and 26–30 month lags [74].

The context of climate change is critical. Bangladesh is highly vulnerable to climate change impacts, including rising temperatures, increasingly erratic and intense rainfall, floods, and shifts in seasons [23]. These changes are creating more favorable conditions for Aedes mosquitoes, potentially extending the transmission season and expanding the geographic areas suitable for vector proliferation and dengue transmission [1]. Projections suggest a significant increase in future dengue cases in Dhaka attributable to temperature rise alone, assuming no adaptation [75]. The strong correlation between observed climate trends in Bangladesh and the recent shifts in dengue epidemiology (earlier/intense outbreaks, geographic spread) provides compelling evidence that climate change is not merely a future risk multiplier but an active amplifier of the current dengue crisis [9].

### 4.3 Socio‐Demographic and Behavioral Drivers

Beyond environmental factors, human societal structures and behaviors significantly shape dengue transmission dynamics. Rapid and often unplanned urbanization is a major driver globally and in Bangladesh [1, 23, 68, 75]. Urban growth leads to high population density, increased human–mosquito contact, inadequate water supply and sanitation infrastructure (forcing water storage practices that create breeding sites), poor waste management, and abundant artificial habitats suitable for Aedes breeding [11]. Studies confirm a positive correlation between population density and dengue incidence in Bangladesh [25].

Human mobility is another critical factor, particularly in disseminating the virus geographically [4]. Increased travel and transportation networks connect urban centers with regional towns and rural areas. Mass population movements during festivals like Eid have been specifically implicated in facilitating the spread of dengue from epicenters like Dhaka to other parts of the country [15]. Conversely, restrictions on movement during the COVID‐19 pandemic were associated with a reduction in dengue incidence in 2020, further highlighting the role of human mobility [76].

At the household level, various risk factors influence infestation risk. Water storage practices necessitated by unreliable piped water supply create numerous breeding sites [26]. Housing characteristics, such as construction type (e.g., slums vs. permanent structures), presence of shaded areas outdoors, and overall socioeconomic status, can affect the availability of breeding sites and exposure risk [57]. Community knowledge, attitudes, and practices (KAP) regarding dengue are also crucial [26]. While awareness of dengue may be high, specific knowledge about vector identification, breeding habits, and effective prevention methods is often lacking, particularly in rural or lower socioeconomic groups. Perceptions of personal risk or susceptibility can be low even when perceived severity is high, leading to unsatisfactory adoption of preventive behaviors like eliminating breeding sites or using personal protection [57].

### 4.4 The Critical Challenge of Insecticide Resistance

Vector control programs in Bangladesh, like many parts of the world, have heavily relied on chemical insecticides to target adult mosquitoes and larvae [39]. Pyrethroids, such as permethrin and deltamethrin, have been the most commonly used class due to their relatively high efficacy against insects and lower toxicity to mammals, often applied through thermal fogging or space spraying. However, the effectiveness of this strategy is severely undermined by the development and spread of insecticide resistance in Aedes populations. Studies conducted in Dhaka and other districts of Bangladesh have documented widespread and high‐intensity resistance to pyrethroids in the primary vector, Ae. Aegypti [77]. Bioassays using standard diagnostic doses (and even multiples thereof) showed very low mortality rates for permethrin (e.g., 2%–24% mortality) and significantly reduced mortality for deltamethrin (e.g., 48%–94% mortality at 10 × dose) in Dhaka populations [78]. Resistance to permethrin has also been detected in Ae. albopictus populations. While susceptibility appears to be largely retained for the organophosphate malathion and the carbamate bendiocarb in tested populations, reliance on these alternatives may also lead to resistance development over time [77].

Resistance in Bangladeshi Ae. aegypti is driven by multiple mechanisms acting concurrently. Target‐site resistance, specifically mutations in the voltage‐gated sodium channel gene (the site of action for pyrethroids), is prevalent. High frequencies of the V1016G knockdown resistance (kdr) mutation, often in homozygous form, have been detected, sometimes co‐occurring with the F1534C mutation, which can further enhance resistance [78]. Alongside this, metabolic resistance mechanisms are also active. Biochemical assays have shown significantly elevated activity of detoxification enzymes, including cytochrome P450 monooxygenases (oxidases), esterases, and glutathione S‐transferases, which can break down or sequester insecticides before they reach their target site [78].

The operational consequences of this intense, multi‐mechanism resistance are significant. Experiments simulating standard pyrethroid aerosol applications in controlled environments showed that a large proportion (up to 74%) of resistant Ae. aegypti from Dhaka colonies survived exposure [78]. This strongly suggests that current pyrethroid‐based interventions, particularly fogging, are likely failing to effectively reduce vector populations in the field and are therefore insufficient for controlling dengue transmission [77]. The presence of multiple resistance mechanisms indicates a robust resistance profile that may adapt quickly to changing insecticide pressures. This makes merely switching between existing chemical classes a potentially short‐lived solution [78]. It powerfully argues for an urgent shift towards integrated vector management strategies that de‐emphasize broad‐scale chemical application and incorporate non‐chemical methods, insecticide resistance management tactics (like rotation or synergists), and novel approaches such as the use of Wolbachia‐infected mosquitoes [79].

## 5 Public Health Response and Interventions

Bangladesh's public health system has grappled with recurrent dengue outbreaks through various response mechanisms, including surveillance, vector control, healthcare provision, community engagement efforts, and, more recently, the development of a national strategic plan. However, these responses have faced significant limitations and challenges, particularly exposed during the large‐scale epidemics.

### 5.1 Surveillance Systems: Structure and Limitations

The national dengue surveillance system is primarily managed by the DGHS, specifically its Management Information System (MIS) unit and the Health Emergency Operation Center, in collaboration with the Institute of Epidemiology, Disease Control and Research (IEDCR) [80]. The system largely relies on passive, hospital‐based reporting, collecting data on laboratory‐confirmed (typically NS1 or IgM positive) dengue cases admitted to designated public and private hospitals, particularly in Dhaka and increasingly from district and sub‐district (Upazila) level facilities [81]. IEDCR also conducts specialized surveillance, including event‐based surveillance using hotlines and media monitoring, and some virological surveillance (serotyping/genotyping) on limited samples [26]. Daily situation reports are often disseminated during outbreaks [1].

Despite these structures, the surveillance system suffers from significant weaknesses that limit its effectiveness for early warning and response guidance [1]. Its passive, hospital‐centric nature means it primarily captures moderate to severe cases requiring hospitalization, missing the vast majority of mild, asymptomatic, or subclinical infections occurring in the community [4, 82]. This leads to a substantial underestimation of the true disease incidence and transmission intensity. Historically, reporting was heavily biased towards Dhaka, providing an incomplete picture of national spread, although this has improved somewhat with expanded reporting from districts [1]. There is a lack of robust, systematic community‐based surveillance to detect transmission trends earlier [83]. A critical gap exists in routine, widespread serotype and genotype surveillance; while some serotyping is done [9], the data is often limited, delayed, and geographically restricted, preventing a real‐time understanding of viral dynamics and population susceptibility crucial for predicting outbreak severity [4]. Entomological surveillance (monitoring vector populations and breeding sites) is also often inadequate or not well integrated with epidemiological data [55]. Delays in data aggregation, analysis, and dissemination can further hamper timely responses [1]. Consequently, the current system functions more as a lagging indicator, documenting outbreaks after they have already escalated, rather than an effective early warning system capable of triggering proactive, targeted interventions [19]. The need for strengthening surveillance across multiple domains (epidemiological, entomological, genomic, community‐based) is a recurring recommendation [26].

### 5.2 Vector Control Strategies: Implementation and Effectiveness

Vector control remains the cornerstone of dengue prevention in the absence of widely available vaccines or specific treatments [11, 78]. Strategies employed in Bangladesh typically include chemical control targeting adult mosquitoes, primarily through insecticide fogging (thermal or ULV space spraying) in affected areas, and LSM aimed at eliminating or treating mosquito breeding sites [11]. Community mobilization efforts to encourage household‐level source reduction are also part of the approach [9, 84].

However, the effectiveness of these conventional methods, particularly as implemented in Bangladesh, is questionable and faces major hurdles. Global systematic reviews have found remarkably little high‐quality evidence (e.g., from randomized controlled trials) supporting the effectiveness of outdoor insecticide space spraying (fogging) in reducing dengue transmission, despite it being a common outbreak response worldwide [85]. In Bangladesh, the documented high levels of pyrethroid resistance in Ae. aegypti populations severely compromise the efficacy of fogging operations that rely on these insecticides [78]. LSM, while theoretically effective, often suffers from challenges related to achieving sufficient coverage, sustainability, and community participation [57]. The sheer number and variety of potential breeding sites in dense urban environments make comprehensive source reduction difficult [62]. Consequently, vector control programs in Bangladesh are frequently described as inadequate or ineffective in preventing large outbreaks [11].

While Integrated Vector Management (IVM)—a rational decision‐making process combining multiple tools and approaches based on local evidence—is recommended [86], its practical implementation appears limited. There seems to be a significant gap between recommended best practices, such as targeting identified key breeding containers [62]. This discrepancy may stem from operational constraints, lack of technical capacity, resource limitations, or socio‐political pressures favoring visible interventions like fogging over more labor‐intensive but potentially more sustainable methods like LSM. Novel vector control tools, such as the use of Wolbachia bacteria to block viral transmission or suppress mosquito populations, are being explored and show promise based on initial characterization of locally adapted strains, offering potential future alternatives [87].

### 5.3 Healthcare System Preparedness and Capacity

Major dengue outbreaks exert immense pressure on Bangladesh's healthcare system, often exceeding its capacity and exposing critical vulnerabilities [1]. The surge in patients requiring hospitalization, particularly those with severe dengue needing intensive monitoring and management, strains resources across the board.

Hospital Bed Shortages: Particularly during peak outbreak periods, hospitals in major hotspots like Dhaka and Chittagong face critical shortages of beds, including intensive care unit (ICU) beds [1, 9]. This shortage is exacerbated by high population density relative to healthcare infrastructure; even Dhaka, with the highest absolute number of beds, has a very low bed‐to‐population ratio [25].

Limited Peripheral Capacity: Healthcare facilities outside major cities, especially at the district and Upazila levels, often lack adequate infrastructure, equipment (e.g., for managing shock or respiratory distress), diagnostic capabilities, and specialized staff to manage severe dengue cases effectively [1]. This contributes to delays in appropriate care or necessitates risky patient transfers to overburdened central hospitals.

Shortage of Trained Personnel: A lack of sufficient healthcare workers (doctors, nurses) trained and experienced in the specific protocols for managing dengue, particularly severe dengue involving meticulous fluid management and recognition of warning signs, is cited as a significant factor contributing to preventable deaths [19, 26].

Resource Constraints: Potential shortages of essential supplies like intravenous fluids (especially specific types like saline), blood products for managing hemorrhage, and diagnostic kits can occur during large outbreaks [19].

Efforts are made to augment capacity during crises, such as designating specific hospitals or wards for dengue patients [88], and initiatives by DGHS to improve diagnostic capacity and train physicians. However, the recurring nature of large outbreaks overwhelming the system indicates that baseline preparedness remains insufficient, particularly at the district level [1]. These healthcare capacity constraints likely act as critical bottlenecks, directly impacting patient outcomes. When combined with delays in seeking care and the geographic expansion of dengue into less‐resourced areas, the limited ability of the health system to absorb and effectively manage surges in severe cases probably contributes significantly to the high mortality rates observed, particularly the alarming number of early deaths after admission.

### 5.4 Community Engagement and Participation

Studies in Bangladesh have assessed community KAP regarding dengue. Findings generally indicate moderate levels of awareness about the disease and its severity, but significant knowledge gaps often persist regarding specific vector characteristics (e.g., identifying Aedes mosquitoes, their biting times) and key breeding sites [57]. This is particularly true in rural areas and among slum dwellers [89].

A critical challenge lies in translating awareness into consistent preventive action. Many studies report that despite knowing dengue is dangerous, individuals often perceive their personal risk or susceptibility as low. Consequently, the adoption of recommended preventive practices, such as regularly searching for and eliminating stagnant water in and around homes, using repellents, or screening windows, remains unsatisfactory among large segments of the population, even among those with higher education levels [57]. Factors influencing preventive behaviors include educational attainment, socioeconomic status, previous personal or family history of dengue, perceived self‐efficacy (belief in one's ability to perform the action), and trust in information sources or authorities [57]. The role of community leaders and religious figures is also considered important in mobilizing action [90].

While community participation in clean‐up campaigns or other initiatives is sometimes observed, achieving sustained behavioral change and high levels of active, ongoing participation remains difficult [57]. Some suggest that stronger measures, such as enforcement of regulations or providing incentives, might be necessary to overcome inertia or perceived barriers [91]. The persistent gap between knowledge and practice suggests that traditional information dissemination campaigns alone are insufficient. There is a clear need for more sophisticated community engagement strategies informed by behavioral science principles, addressing specific local barriers, leveraging social networks and trusted leaders, and empowering communities to take ownership of prevention efforts.

### 5.5 National Strategic Plan for Dengue Prevention and Control (2024–2030)

In response to the escalating dengue crisis, particularly the devastating 2023 outbreak, the Government of Bangladesh, through the Ministry of Health and Family Welfare and DGHS, formulated a comprehensive 7‐year dengue prevention and control strategic plan for the period 2024–2030 (Table 2) [93]. This plan represents a formal recognition of the severity of the problem and outlines a comprehensive, multi‐pronged approach aimed at reducing dengue incidence and mortality. Its stated vision is a “Dengue‐free Bangladesh,” with specific targets of achieving an incidence below 100 cases per 100,000 population and a CFR below 0.1% by 2030 [93].

**TABLE 2:** Key objectives and components of the National Dengue Strategic Plan (2024–2030).

Strategic objective area	Key components/activities (summary)	Source
1. Governance & Coordination	Establish central & national coordination committees/task forces; multi‐sectoral involvement; policy recommendations; resource mobilization.	[92]
2. Healthcare Capacity Enhancement	Update clinical guidelines; train doctors/nurses/staff on diagnosis, warning signs, fluid management, ICU care; strengthen lab diagnostics; ensure the availability of resources.	[92]
3. Integrated Vector Management (IVM)	Adopt National IVM strategy; strengthen entomological capacity (staff, training, national lab); routine vector surveillance & mapping; effective insecticide use & resistance monitoring; promote source reduction (e.g., 4S campaign).	[92]
4. Surveillance & Early Warning	Institutionalize nationwide real‐time surveillance (routine, case‐based, community); MIS integration; death investigation (verbal autopsy); data analysis for action; prediction models & GIS mapping for early warning.	[92]
5. Risk Communication & Community Engagement (RCCE)	Form RCCE committees; standardize messages; develop sectoral guidance; support community surveillance; engage private sector; media campaigns; mobilize local government & field workers; use digital media.	[92]
6. Research & Innovation	Research effective vaccines (selection, manufacturing); evaluate new drugs/management protocols; develop innovative surveillance/early warning; study vector behavior/resistance; develop new vector control methods.	[92]
7. Dengue Vaccination	Evaluate & select WHO‐prequalified vaccines; plan delivery rollout; institutionalize monitoring; community awareness & advocacy.	[92]

The plan explicitly emphasizes a multi‐sectoral approach, recognizing that dengue control requires collaboration beyond the health sector. While the formulation of this strategic plan is a crucial and positive development, its ultimate success will depend entirely on its effective implementation. This requires sustained political commitment, adequate and consistent resource allocation, strong leadership, and the capacity to overcome the deep‐rooted systemic weaknesses in surveillance, vector control, healthcare delivery, and community engagement that the plan itself aims to address. Given the history of recurrent large outbreaks despite previous efforts, translating this strategy into tangible, sustained improvements on the ground represents the most significant challenge.

## 6 Research Gaps and Lessons Learned

The escalating dengue crisis in Bangladesh, particularly the severe outbreaks of recent years, highlights critical gaps in knowledge and underscores important lessons for future prevention and control efforts. Addressing these gaps and internalizing these lessons is paramount for developing more effective and resilient strategies.

### 6.1 Identifying Key Knowledge Gaps

Despite increased attention, significant gaps remain in the understanding and management of dengue in Bangladesh:

True Disease Burden: The reliance on passive, hospital‐based surveillance systems means the actual incidence of dengue, including the large burden of mild, subclinical, and asymptomatic infections in the community, remains poorly quantified. Improved methodologies, potentially including community‐based cohort studies or enhanced serosurveys, are needed for accurate burden estimation [15].

Viral Genomics and Evolution: There is a significant lack of comprehensive, nationwide data on the circulating DENV genotypes, their specific characteristics (e.g., virulence potential), and evolutionary dynamics within Bangladesh. Understanding viral genetic factors driving transmission intensity or severity is crucial but currently limited [4].

Determinants of Severity and Mortality: While secondary infection is a known risk factor, a deeper understanding of the specific host, viral, and immunological factors driving the high CFR, the phenomenon of rapid death post‐admission, and the observed demographic disparities (e.g., higher female mortality) is urgently needed [82]. The role of co‐morbidities and potential interactions with other infections like COVID‐19 requires further exploration [19].

Vector Ecology and Competence: More research is needed on the relative roles and competence of Ae. aegypti and Ae. albopictus in different ecological settings (urban, peri‐urban, rural), precise identification and quantification of productivity from different container types (“key containers”) across diverse settings, and factors influencing vector survival and behavior [15, 60].

Intervention Effectiveness: There is a striking lack of rigorous, context‐specific evidence, particularly from randomized controlled trials, evaluating the effectiveness of commonly used vector control interventions like insecticide fogging in Bangladesh, especially given the high levels of resistance [85]. Similarly, the effectiveness of different community engagement strategies needs robust evaluation.

Climate Change Impacts: While the link between climate and dengue is established, refining predictive models that incorporate local climatic variability, land use changes, and socio‐ecological factors is necessary for accurate forecasting and adaptation planning [67].

Diagnostics: Development and validation of improved rapid diagnostic tools, potentially including point‐of‐care tests capable of serotype identification, are needed, especially for resource‐limited settings.

Vaccines and Therapeutics: Local data on the efficacy, safety, cost‐effectiveness, and optimal implementation strategies for potential dengue vaccines (e.g., Dengvaxia, Qdenga) are essential before considering widespread introduction [94, 95]. Research into effective antiviral therapeutics remains a global priority [51].

### 6.2 Lessons From Recent Outbreaks

The devastating outbreaks of 2019 and especially 2023 and 2024 offer stark lessons:

Proactive Preparedness is Essential: The largely reactive nature of responses to outbreaks has proven insufficient [1]. Effective preparedness requires robust early warning systems based on integrated surveillance, pre‐defined response triggers, and pre‐positioned resources, rather than scrambling to react once an epidemic is underway.

Dengue is a Nationwide Problem: Control strategies narrowly focused on historical urban epicenters like Dhaka are no longer adequate. Dengue has demonstrably spread nationwide, including into rural areas, necessitating geographically comprehensive and context‐specific interventions [4].

Systemic Weaknesses Must Be Addressed: The recent epidemics brutally exposed deep‐seated weaknesses across the public health system: inadequate surveillance for early detection and monitoring, vector control hampered by resistance and ineffective methods, healthcare capacity insufficient to handle surges in severe cases (leading to high mortality and rapid deaths post‐admission), and community engagement efforts failing to achieve sustained preventive behaviors [1].

Data Drives Effective Response: The lack of timely, comprehensive, and integrated data (including community case burden, serotype/genotype distribution, vector density/resistance, intervention coverage/impact) severely hinders the ability to understand outbreak dynamics, target interventions effectively, and adapt strategies in real‐time [4].

Multi‐Sectoral Collaboration is Non‐Negotiable: Dengue control cannot be solely the responsibility of the health ministry. Effective prevention requires coordinated action involving local government bodies (responsible for waste management, urban planning, and local vector control), water authorities, education sectors, environmental agencies, private sector actors, and communities themselves [96].

Perhaps the most critical lesson is the apparent failure to fully internalize and act upon lessons from previous outbreaks. The recurrence of massive epidemics despite prior experience, and the persistence of known systemic weaknesses (e.g., continued reliance on likely ineffective pyrethroid fogging despite known resistance, recognized surveillance gaps), suggest significant barriers—be they political, financial, or operational—are preventing the translation of knowledge into sustained, effective action and adaptation. Overcoming these implementation barriers is as crucial as identifying the technical solutions.

## 7 Future Directions and Recommendations

Based on the synthesized evidence and identified challenges, a multi‐faceted and sustained effort is required to effectively combat the escalating dengue crisis in Bangladesh. Future directions and recommendations should focus on strengthening foundational systems, adopting innovative approaches, and fostering robust collaboration.

### 7.1 Strengthening Surveillance and Early Warning

A paradigm shift from passive reporting to proactive, integrated surveillance is essential. This includes:

Implementing Integrated Surveillance: Combining real‐time epidemiological data (hospital and community‐based) with entomological surveillance (vector density, breeding sites, resistance monitoring), serological surveillance (population immunity profiles), and genomic surveillance (tracking viral serotypes/genotypes) to provide a holistic understanding of transmission dynamics [9, 97]. Establishing sentinel sites for comprehensive, longitudinal monitoring could be valuable.

Enhancing Laboratory Capacity: Building and sustaining nationwide laboratory capacity for timely and accurate diagnosis, including widespread access to RDTs, and critically, scaling up capacity for routine DENV serotyping and genotyping at regional or national reference laboratories [1, 4].

Developing Predictive Models: Leveraging integrated surveillance data along with climate/environmental variables and human mobility data to develop and validate robust forecasting models capable of providing early warnings (weeks to months in advance) of potential outbreaks at national and sub‐national levels [23].

### 7.2 Advancing Integrated Vector Management (IVM)

Moving beyond reliance on potentially ineffective chemical control requires a commitment to true IVM:

Effective IVM Implementation: Ensuring the National IVM strategy [93] is fully implemented with adequate resources, technical expertise, and monitoring. This involves evidence‐based selection and combination of control tools tailored to local contexts.

Managing Insecticide Resistance: Implementing routine, systematic monitoring of insecticide resistance in key vector populations across the country. Developing and operationalizing resistance management strategies, such as rotating insecticide classes, using mixtures or mosaics, and potentially employing synergists like piperonyl butoxide (PBO) where appropriate [88]. Reducing reliance on pyrethroids is critical.

Optimizing Larval Source Management (LSM): Focusing LSM efforts on identified “key” breeding containers through targeted interventions. Ensuring community participation and developing sustainable models for routine source reduction, moving beyond sporadic clean‐up campaigns [57]. Rigorous evaluation of the LSM program effectiveness is needed.

Exploring and Scaling Novel Tools: Investing in the evaluation and potential scale‐up of innovative vector control methods like Wolbachia‐based strategies (population suppression or replacement), which have shown promise in trials and local strain characterization [87]. Assessing the feasibility of other approaches, like the Sterile Insect Technique or genetically modified mosquitoes.

Strengthening Entomological Capacity: Building a skilled workforce of entomologists and vector control technicians at national and local levels, supported by adequate infrastructure for vector surveillance and research [93].

### 7.3 Enhancing Healthcare Capacity and Clinical Management

Strengthening the healthcare system's ability to manage dengue patients, especially during surges, is vital for reducing mortality:

Boosting Healthcare Infrastructure: Strategically increasing hospital bed capacity, particularly ICU beds equipped for managing severe dengue, in both urban centers and peripheral areas identified as high‐risk or underserved. Ensuring reliable supply chains for essential diagnostics, intravenous fluids, blood products, and medications [4].

Improving Clinical Management: Regularly updating and widely disseminating national dengue clinical management guidelines based on the latest evidence. Implementing comprehensive, ongoing training programs for doctors, nurses, and other healthcare staff at all levels, focusing on early recognition of warning signs for severe dengue, appropriate fluid management protocols (crucial for preventing shock and fluid overload), and management of complications [39]. Establishing clinical audit systems to monitor adherence to guidelines and patient outcomes.

Researching Treatment Protocols: Supporting clinical research to evaluate novel therapeutic approaches and optimize management strategies for severe or atypical dengue presentations [93].

### 7.4 Improving Community Engagement and Risk Communication

Transforming community engagement from passive information dissemination to active partnership is key:

Behaviorally‐Informed RCCE: Designing and implementing Risk Communication and Community Engagement strategies grounded in behavioral science principles, identifying and addressing specific local barriers and motivators for preventive actions (source reduction, personal protection, timely care‐seeking) [102]. Tailoring messages and approaches for different demographic groups and settings (urban/rural).

Empowering Communities: Fostering genuine community participation and ownership through collaborative approaches, potentially involving community health workers, volunteers (including youth), local committees, and exploring the use of incentives or participatory monitoring tools [90].

Strengthening Multi‐sectoral Collaboration: Building effective partnerships between health authorities, local government institutions, NGOs, schools, religious leaders, and community groups to ensure coordinated and sustained community mobilization efforts [96].

### 7.5 Addressing Climate Change

Integrating climate change considerations into dengue control is increasingly necessary:

Climate‐Informed Planning: Explicitly incorporating climate change projections and vulnerability assessments into long‐term dengue prevention and control strategies [82].

Climate‐Based Early Warning: Utilizing climate data and forecasts to enhance surveillance sensitivity and improve the accuracy and lead time of outbreak prediction models [23].

### 7.6 Pursuing Vaccination and Therapeutics

While vector control remains primary, vaccines and treatments offer long‐term hope:

Evaluating Vaccines: Carefully evaluating the suitability, safety, efficacy, and cost‐effectiveness of WHO‐prequalified dengue vaccines (e.g., Dengvaxia, Qdenga) within the specific epidemiological context of Bangladesh before any decision on introduction [93]. If adopted, developing robust pre‐vaccination screening strategies (if required) and comprehensive implementation and monitoring plans. Notably, Bangladesh has not yet introduced any dengue vaccine into its national immunization programme; consequently, no domestic post‐vaccination surveillance or vaccine adverse event data currently exists. Generating such local safety, immunogenicity, and effectiveness data through well‐designed pre‐introduction studies represents a critical and urgent research priority.

Supporting Therapeutics Research: Contributing to or closely monitoring global research efforts aimed at developing specific antiviral drugs for dengue [51].

### 7.7 Adopting a One Health Approach

Recognizing the interconnectedness of human, animal (vector), and environmental health is crucial:

Promoting Inter‐sectoral Collaboration: Fostering genuine collaboration and joint planning between ministries and agencies responsible for health, environment, urban planning, water resources, waste management, and agriculture to address the upstream determinants of dengue transmission in a holistic manner [19].

## 8 Conclusion

Dengue fever has transformed from an intermittent public health concern into a severe, nationwide crisis in Bangladesh. The period since 2000 has been marked by exponential growth in outbreak frequency, magnitude, and geographic scope, culminating in the unprecedented 2023 epidemic. This review highlights the critical interplay of serotype dynamics, climate change, rapid urbanization, insecticide resistance, and systemic gaps in surveillance, healthcare, and community engagement that collectively amplify this crisis. The National Strategic Plan for Dengue Prevention and Control (2024–2030) provides a necessary and comprehensive framework; however, its success depends on overcoming persistent implementation barriers. Sustained political commitment, multi‐sectoral coordination, evidence‐based Integrated Vector Management, strengthened multi‐domain surveillance, and genuine community partnership—embedded within a One Health framework—are essential for reversing the current trajectory and achieving meaningful, long‐term dengue control in Bangladesh.

## Author Contributions

**Md. Jubayer Hossain:** conceptualization, investigation, writing – original draft, writing – review and editing, validation, methodology, formal analysis, project administration, resources, supervision, data curation. **Nishat Afrin Mim:** validation, writing – review and editing, investigation. **Nargees Akter:** investigation, validation, writing – review and editing. **Soniya Akter Sony:** investigation, validation, writing – review and editing. **Ekramul Haque Saikat:** validation, writing – review and editing, investigation. **Md. Al‐Amin Hossain:** investigation, validation, writing – review and editing. **Nusrat Parvin:** investigation, validation, writing – review and editing. **M. R. Evan:** investigation, validation, writing – review and editing. **Jayma Jannat Juma:** investigation, validation, writing – review and editing. **Progga Parmita Roy:** investigation, writing – review and editing, validation. **Md. Fakhrul Islam Maruf:** validation, writing – review and editing, investigation. **Rayhan Islam:** validation, writing – review and editing, investigation. **Abrar Labib:** investigation, validation, writing – review and editing. **Zayeda Akter Shatabde:** investigation, validation, writing – review and editing. **Manisha Das:** investigation, validation, writing – review and editing. **Md Amanulla:** investigation, validation, writing – review and editing.

## Funding

The authors have nothing to report.

## Disclosure

All authors have read and approved the final version of the manuscript. Md. Jubayer Hossain had full access to all of the data in this study and takes complete responsibility for the integrity of the data and the accuracy of the data analysis.

## Ethics Statement

The study is based on secondary data and published literature; no human participants or patient data were directly involved.

## Conflicts of Interest

The authors declare no conflicts of interest.

## Preprint and Conference Disclosure

This manuscript has not been posted as a preprint and has not been presented at any scientific conference or seminar.

## AI Usage Disclosure

This manuscript was prepared by the listed human authors, who take full responsibility for its content. Large Language Models (LLMs), specifically ChatGPT‐5 (OpenAI), were used solely to assist in improving the clarity, grammar, and flow of the language. The authors reviewed and edited all AI‐generated text to ensure accuracy, integrity, and adherence to scientific standards. No content, data interpretation, or conclusions were generated autonomously by the AI tool.

## Transparency Statement

Md. Jubayer Hossain affirms that this manuscript is an honest, accurate, and transparent account of the study being reported; that no important aspects of the study have been omitted; and that any discrepancies from the study as planned have been explained.

## Data Availability

All data and materials used in this review are drawn from published sources, official reports, and publicly available databases. Detailed references are provided in the manuscript.
